# Development and preclinical testing of the critical care pain observation tool for family caregiver use (CPOT‐Fam)

**DOI:** 10.1002/hsr2.986

**Published:** 2022-12-09

**Authors:** Anmol Shahid, Bonnie G. Sept, Shelly Longmore, Victoria S. Owen, Stephana J. Moss, Andrea Soo, Kirsten M. Fiest, Céline Gélinas, Henry T. Stelfox

**Affiliations:** ^1^ Department of Critical Care Medicine, Cumming School of Medicine University of Calgary & Alberta Health Services Calgary Alberta Canada; ^2^ Department of Psychiatry, Hotchkiss Brain Institute Cumming School of Medicine Calgary Alberta Canada; ^3^ Department of Community Health Sciences University of Calgary Calgary Alberta Canada; ^4^ Centre for Nursing Research and Lady Davis Institute, Ingram School of Nursing, Jewish General Hospital—CIUSSS West‐Central Montreal McGill University Montreal Canada; ^5^ O'Brien Institute for Public Health University of Calgary Calgary Alberta Canada

**Keywords:** critical care pain observation tool, family partnership, intensive care unit pain, pain assessment, quality improvement, tool development

## Abstract

**Background and Aims:**

Pain assessment in noncommunicative intensive care unit (ICU) patients is challenging. For these patients, family caregivers (i.e., family members, friends) may be able to assist in pain assessment by identifying individualistic signs of pain due to their intimate patient knowledge. This study adapted the critical care pain observation tool (CPOT) to facilitate pain assessment in adult ICU patients by family caregivers.

**Methods:**

This study was conducted through three distinct phases:

(1) CPOT adaptation for family caregiver use (to create the CPOT‐Fam): A working group met monthly to adapt the CPOT and develop educational material and sample cases for practice scoring until consensus was reached.
(2) CPOT‐Fam preclinical testing: Family caregiver study participants viewed educational materials and scored four randomly selected sample cases using the CPOT‐Fam. Scores were compared to reference scores to assess agreement and identify CPOT‐Fam sections requiring revision. Open‐ended feedback on the CPOT‐Fam was collected.
(3) CPOT‐Fam revision: the CPOT‐Fam was revised by the working group considering score agreement and feedback received from study participants.

**Results:**

Of the *n* = 30 participants, *n* = 14 (47.0%) had experience with an ICU patient. Agreement between CPOT‐Fam participant scores and reference scores were highest for the *vocalization* dimension (*Is the patient making any sounds?*; Intraclass correlation coefficient; ICC = 1.0) and lowest for the *body movements* dimension (*What are the patient's body movements like?*; ICC = 0.85. Participants indicated they found the CPOT‐Fam to be “informative” and “easy‐to‐use” but “not graphic enough”; participants also indicated that descriptors like “lack of breath” and “struggling to move” are helpful with identifying individualistic behaviors of pain exhibited by their loved ones.

**Conclusion:**

The CPOT‐Fam shows ease of use and may be of value in involving family caregivers in ICU care. Clinical pilot testing is needed to determine feasibility and acceptability and identify further areas for refinement.

## 1 INTRODUCTION

Pain is commonly experienced by patients in the intensive care unit (ICU).^1^, ^2^ As self‐reporting is the gold standard measure of pain, alternative assessment methods are required for patients who are unable to self‐report their pain according to their clinical condition and cognitive capacity.^3^, ^4^ For these patients, proxy reporters (e.g., parents, children, spouses, professional caregivers) can assist in pain recognition as these individuals are often familiar with the patient's behavior and can pinpoint small changes in patient conduct indicative of pain.^2^, ^4^, ^5^ Hospitalized patients' self‐reported pain ratings have been reported to be closer in agreement with family caregiver proxy reporters pain ratings rather than ratings by nurses and physicians.^5^, ^6^ Therefore, involving family caregivers in pain assessment of patient's unable to self‐report may allow for earlier pain recognition, reduce anxiety, and improve satisfaction with care for patients and their loved ones.^7^

Of the available pain assessment tools suitable for critically ill patients unable to self‐report, the critical care pain observation tool (CPOT) is recommended for clinical use by practice guidelines and widely used.^8^, ^9^ The CPOT is comprised of four behavioral items (i.e., categories) to allow assessment of pain in critically ill patients: (1) *facial expression*; (2) *body movements*; (3) *compliance with ventilator* (if intubated) or *vocalization* (if not intubated); and (4) *muscle tension*.^10^, ^11^ Each item can be scored from 0 to 2 (0 being the lowest score); the scores are additive for a maximum score of 8.^12^ The CPOT has shown moderate correlations (0.59 and 0.71; *p* < 0.05) to self‐reported pain intensity during nociceptive procedures in the ICU (such as turning) and reasonable sensitivity (86%) and specificity (78%) when used by ICU clinicians in ventilated and non‐ventilated ICU patients.^8^, ^11^, ^13^ While the CPOT has been developed for use by clinicians, it has shown suitability for use by proxy reporters of pain (hereon referred to as family caregivers to describe family members and friends).^10^

When used by family caregivers given sufficient training, the CPOT has been reported to empower family caregivers to: (1) confirm their observations of pain behaviors, (2) allow more focus on the patient, and (3) advocate for better pain management.^10^ However, many family caregivers using the CPOT have not found all dimensions of the tool relevant to their understanding of pain.^10^ This may be due to complex terminology describing scoring options in the CPOT, written for use by ICU clinicians.

To address the need for a pain assessment tool suitable for family caregiver use in the ICU, this study aimed to adapt the CPOT for family use and test the tool preclinically to determine its appropriateness and areas for revision.

Specifically, the main objectives for this study were to:

(1) Modify the content of the CPOT to be suitable for use by family caregivers (creating the CPOT‐Fam); and
(2) Complete preclinical testing of the CPOT‐Fam using sample cases to determine agreement in scores between family caregivers and research staff, and collect feedback from participants on the CPOT‐Fam; and
(3) Revise the CPOT‐Fam with feedback received from study participants and research team members to prepare the tool for a future clinical pilot study.

## 2 METHODS

This study was executed through three distinct phases, described in detail below. Permission to adapt the CPOT was sought from AACN (American Association of Critical Care Nurses) and approval was received to adapt the English language version of the tool for family caregiver use. Ethical approval to conduct this study was obtained from the University of Calgary Conjoint Health Research Ethics Board (REB 21‐0748) alongside a research agreement with the health custodian at Alberta Health Services

### 2.1 Phase 1: Development

#### 2.1.1 Creating the CPOT‐Fam

To discuss adaptation of the CPOT for family‐caregiver use (i.e., creating the CPOT‐Fam) and address the knowledge to practice gap, a working group of researchers, ICU clinicians, and patient partners with critical care research experience was conferred. Group members were selected based on their prior experience (clinical ICU care, clinical research, patient partner). The group discussed simplification of CPOT dimension content to suit family caregiver use and agreed that the CPOT‐Fam should be paired with educational materials (to provide family caregiver users with information on pain and information on how to use the tool), and sample cases (to allow practice scoring). With feedback from the working group meetings, the research lead drafted the CPOT‐Fam and iteratively revised the tool and accompanying materials alongside patient partners in anticipation for preclinical testing (detailed below).

#### 2.1.2 Creating educational materials and sample cases

A subworking group including the lead researcher, patient partners, and a ICU nurse created educational materials to accompany the CPOT‐Fam in a digital format. Educational materials included (1) information on ICU pain; (2) information on how and when to use the CPOT‐Fam and; (3) sample cases to facilitate family CPOT‐Fam scoring. The educational material was prepared at a sixth‐grade reading level with guidance from the Patient Education Materials Assessment Tool for print materials (PEMAT‐P)^14^ to ensure that individuals with varying educational backgrounds could understand and act upon the information presented. Sample cases was created to reflect each possible scoring combination on the original CPOT (162 unique scoring combinations, 81 for intubated patients, and 81 for non‐intubated patients).^12^ Clinically unlikely scoring combinations (e.g., an intubated patient who had a score of 0 on *facial expression* and a score of 2 on *compliance with ventilator*) were identified through team discussion and excluded, leaving 120 sample cases for use in the preclinical testing phase.

### 2.2 Phase 2: CPOT‐Fam preclinical testing

The CPOT‐Fam, accompanying educational materials, and sample cases were tested preclinically (shown in Supporting Information: Appendix 1).

#### 2.2.1 Study setting, participants, and recruitment

Study participants (including family‐caregivers of current or former ICU patients and interested members of the public) were recruited virtually using social media (e.g., Twitter channels) and Bethecure.ca (a resource providing members of the general public information on participating in research and available opportunities), as well as in person at the Foothills Medical Center medical‐surgical ICU in Calgary, Canada between October 15 and December 15, 2021. Participants were eligible for inclusion in this study if they were over 18 years of age, able to communicate in English (i.e., understand, read, speak), and able to provide informed consent. To minimize risk of COVID‐19 exposure to participants, study materials were provided to participants digitally whenever possible. Consent was obtained verbally (over the telephone) using a recruitment script that ensured potential participants were provided adequate information about the study and understood their role.

#### 2.2.2 Procedures

Study participants were assigned a unique identification number and provided an online link to view educational materials. Participants were then administered an online, Qualtrics, survey where they were asked to sequentially: (1) assign CPOT‐Fam scores to four randomly allocated sample cases, (2) complete a CPOT‐Fam follow‐up survey, and (3) provide demographic information. The research team facilitated participants' completion of the educational materials, sample cases, the demographic survey, and the follow‐up survey by being present to answer questions by telephone or email. All data was stored securely on a University of Calgary‐approved digital location in accordance with institutional procedures. Study procedures and instruments used are described in further detail in a peer‐reviewed protocol manuscript.^15^

#### 2.2.3 Data analysis

Quantitative data collected from participants was analyzed using Microsoft Excel. Participant characteristics and responses to closed‐ended questions (e.g., multiple choice, yes/no) were compiled into counts and percentages. The intraclass correlation coefficient (ICC) was used to determine agreement and degree of correlation between participant generated CPOT‐Fam scores and reference CPOT‐Fam scores for all sample cases (ICC determined for each CPOT‐Fam question score and total CPOT‐Fam score) before being interpreted according to accepted recommendations.^16^ The ICC was calculated using Microsoft Excel (version 2207; Microsoft Corporation).

Textual data collected on the follow‐up survey (i.e., feedback on the CPOT‐Fam) was analyzed using NVIVO 12 (QSR International). A qualitative content analysis was completed by two research team members using an inductive sentiment approach. Research team members independently reviewed the complete data set and identified initial codes that represented categories of feedback received from study participants. The research team members then compared individually identified codes and discussed converging and diverging ideas before finalizing the codebook. All data were coded independently in duplicate using the finalized codebook.

#### 2.2.4 Sample size and power considerations

Participants were recruited according to an estimate of ICC = 0.85 between participant‐assigned CPOT‐Fam scores and reference scores (i.e., researcher‐assigned scores). To estimate a 95% confidence interval with a width of 0.099 (±0.050), *n* = 120 sets of scores from *n* = 30 subjects were required (in which each subject scored 4 sample cases).

### 2.3 Phase 3: CPOT‐Fam revision

The research lead modified the CPOT‐Fam in consideration of feedback received during the preclinical testing phase and presented the revised version to the working group (including CJ, RP). The CPOT‐Fam was then further discussed with the working group using a modified nominal group technique^17^, ^18^ to ensure the group was satisfied with the wording used in each dimension of the CPOT‐Fam and the tool's suitability for future clinical pilot testing.

## 3 RESULTS

### 3.1 Development of the CPOT‐Fam

The working group held four meetings between December 2020 and May 2021. The group determined that terminology used in the original CPOT would be challenging for users of varying educational backgrounds and health literacy to utilize and major revisions would be needed to adapt the tool for family caregiver use. The working group agreed that as the CPOT is a well‐validated tool commonly used in ICUs, it would be important to retain the original dimensions (i.e., (1) *facial expression*; (2) *body movements*; (3) *compliance with ventilator* (if intubated) or *vocalization* (if not intubated); and (4) *muscle tension*) and scoring system (i.e., 0−2 score on each dimension).^10^, ^11^

The lead researcher and patient partners built the CPOT‐Fam by first expressing CPOT tool dimensions in question format to increase user‐friendliness. For example, the *body movements* CPOT dimension was posed as the following question in the CPOT‐Fam: “*What are the patient's body movements like?*.” Scoring options for each CPOT dimension were also simplified to reflect language that could be understood by individuals without a specific health or educational background. For example, a score of 0 on the *body movements* dimension is described as “absence of movements or normal position” in the original CPOT. In the CPOT‐Fam, this scoring description was modified to “relaxed or comfortable (examples: lying down, sitting, or moving without pain).” The CPOT‐Fam was then presented to the sub‐working group and two rounds of major revisions and multiple rounds of minor revisions were completed before the group deemed the CPOT‐Fam tool ready for preclinical testing.

### 3.2 Preclinical test of the CPOT‐Fam

Of the 35 individuals who expressed interest in participating, most (*n* = 30; 86.0%) completed participation in the study. The majority of participants were male (*n* = 20; 67.0%), between 20 and 39 years of age (*n* = 23; 77.0%), college or university educated (*n* = 18; 60.0%), and belonged to an English‐speaking household (*n* = 22, 73.0%). Fourteen participants (47.0%) reported a personal connection to someone currently or previously in ICU. Participant characteristics are shown in detail in Table 1.

**Table 1:** Characteristics of participants from preclinical testing phase

Characteristics	Participants (*n* = 30)
Current or previous relationship with ICU patient, *n* (%)	14 (47.0%)
Age (years), *n* (%)	
<20	1 (3.33%)
20−39	23 (77.0%)
40−59	3 (10.0%)
60+	3 (10.0%)
Male, *n* (%)	20 (67.0%)
Education, *n* (%)	
Highschool diploma or less	3 (10.0%)
Vocational/trade certification	0 (0.0%)
Some college or university	4 (13.3%)
College or university degree	18 (60.0%)
Higher education or professional degree	5 (16.7%)
Language spoken at home, *n* (%)	
English	22 (73.3%)
Other	7 (23.3%)
Prefer not to say	1 (3.33%)

Abbreviation: ICU, intensive care unit.

#### 3.2.1 Agreement in CPOT‐Fam scoring of sample cases

Agreement between CPOT‐Fam reference scores and participant scores on sample cases was high (defined as ICC ≥0.81)^16^ for all CPOT‐Fam scoring dimensions (shown in Table 2).^19^ Agreement was highest for the *compliance with ventilator* or *vocalization* dimension, consisting of Questions 2a (*How is the patient breathing?*; ICC = 0.97) and 2b (*Is the patient making any sounds?*; ICC = 1). Dimensions with reduced agreement were the *body movements* and *muscle stiffness* dimensions where Question 4 (*What are the patient's body movements like?*; ICC = 0.85) had the lowest agreement. The breakdown of agreement by each CPOT‐Fam dimension is shown in Table 2.

**Table 2:** Participant score agreement with reference standards

CPOT‐Fam scoring categories	Percent agreement (%)^a^	Intraclass correlation coefficient^a^
Q1 Is the patient breathing through a breathing tube or breathing machine? (Yes/no score)	98.3	‐
Q2a How is the patient breathing?	96.6	0.97
*Q2a Displayed if Q1 answer was YES (0−2 score)		
Q2b Is the patient making any sounds?	100.0	1
*Q2b Displayed if Q1 answer was NO (0−2 score)		
Q3 What is the patient's facial expression? (0−2 score)	92.3	0.92
Q4 What are the patient's body movements like? (0−2 score)	88.5	0.85
Q5 Does the patient have stiff muscles? (0−2 score)	92.3	0.92
Total (0−8 score)	78.8^b^	0.92

Abbreviation: CPOT, critical care pain observation tool.

^a^ Percent agreement and intraclass correlation coefficients were generated through comparison of reference scores with participant scores for the listed CPOT‐Fam scoring categories.

^b^ Represents % agreement in total CPOT‐Fam scores (ranging from 0−8).

#### 3.2.2 Participant feedback—closed‐ended

All participants who had a personal connection with a current or previous ICU patient (*n* = 14; 100%) indicted that they would feel empowered to act if they identified pain in their family member. Of these, 10 (71.0%) participants indicated they felt extremely comfortable or somewhat comfortable in their ability to tell whether a loved one was experiencing pain, whereas 4 (28.6%) participants indicated they felt extremely or somewhat uncomfortable in doing this.

#### 3.2.3 Participant feedback—open‐ended

**Figure 1:**
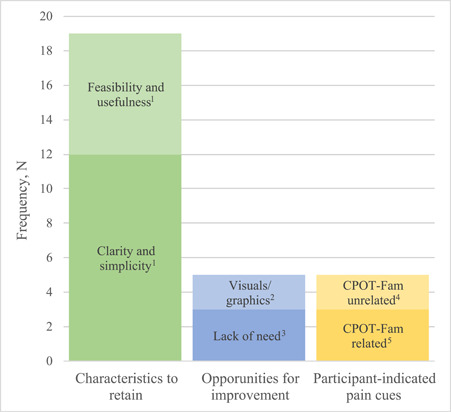
Participant (*n* = 30) feedback on CPOT‐Fam, categorized by feedback content and type. CPOT, critical care pain observation tool.

Twenty‐seven (90.0%) of 30 total participants provided a text response to the open‐ended question prompt: *Please describe your experience with using the pain detection method (i.e., CPOT‐Fam) in a few words*. Text responses were categorized according to feedback content and presented as frequency (*n*) of *characteristics to retain* (*n* = 19), *opportunities for improvement* (*n* = 5), and *participant‐indicated pain cues* (*n* = 5), shown in Figure 1. The feedback around *characteristics to retain* included affirmative and optimistic comments received regarding the *feasibility and usefulness* of the CPOT‐Fam (*n* = 7), and *clarity and simplicity* of the CPOT‐Fam (*n* = 12). The feedback received around *opportunities for improvement* consisted of feedback on *visuals and graphics* (*n* = 2) and *lack of need* (i.e., families don't need a specific tool to identify pain in a patient) (*n* = 3). Some participants wrote about their personal experiences with identifying pain (categorized as *participant‐indicated pain cues*), using terminology and concepts that were *related to existing CPOT‐Fam dimensions* (*n* = 3) or *unrelated to existing CPOT‐Fam dimensions* (*n* = 2). Select quotes from text feedback representing each category are shown below.

“Trying to understand what level of pain someone is going through is difficult, especially if they can't talk for themselves. It's the little twitches that give you clues.”

“Not graphic enough ‐ words are not specific enough open to interpretation.”

“I thought this provided me with a good checklist to run through to detect pain.”

Open‐ended feedback on the CPOT‐Fam was further compiled into actionable categories (shown in Table 3), which included *content‐based feedback* in addition to the existing feedback categories shown in Figure 1. Actionable feedback of note included participants' suggestions to increase graphics and minimize usage of ambiguous or complex words in the CPOT‐Fam. Additionally, actionable participant feedback highlighted CPOT‐Fam content areas needing further modification such as descriptors for the facial expression, body movement, and breathing. Participant feedback also suggested addition of new CPOT‐Fam dimensions that would represent individualistic behaviors of pain exhibited by ICU patients.

**Table 3:** Actionable participant‐derived feedback on CPOT‐Fam (version 1), categorized by characteristics to retain and opportunities for improvement

Actionable feedback categories	Characteristics to retain	Opportunities for improvement
Clarity and simplicity	Retain graphics/illustrations Retain concise, simple language to improve usability by individuals with varying educational and language backgrounds	Minimize usage of complex or unnecessary words Increase frequency of graphics Reduce confusing/ambiguous wording
Content	Retain informative checklist of pain behaviors	Create new CPOT‐Fam pain dimensions: ‐ Create new CPOT‐Fam dimensions to supplement existing dimensions ‐ Incorporate pain behaviors such as twitching, feeling general discomfort, and any personal displays of pain a loved one would be able to identify Expand existing CPOT‐Fam dimensions: ‐ Facial expression (e.g., add looking tired) ‐ Body movements (e.g., add struggling to move) ‐ Breathing (e.g., add difficulty breathing)
Feasibility and usefulness		Enhance CPOT‐Fam relevance across all types of relationships and intimacy levels

Abbreviation: CPOT, critical care pain observation tool.

### 3.3 Revision of the CPOT‐Fam

The working group was assembled once again in January 2022 to discuss actionable feedback received from participants during the preclinical CPOT‐Fam testing. In a structured group meeting, the research lead presented each feedback item to the group and invited comments from each working group member sequentially. The comments were noted onto online note‐taking platform and any duplicate comments were removed. The working group then ranked the suggestions for revision in order of perceived priority. Open‐ended discussion was continued on each of the items until consensus was achieved. The working group determined that for the CPOT‐Fam to be most effective, it would (1) need to be based on the four original dimensions of the original CPOT and (2) need to stand‐alone as a simple tool that would not require any training or educational materials to use. The working group also prioritized restructuring the tool into a flow‐diagram format and adding visual representations of scale elements.

After the working group meeting, the research lead redrafted the CPOT‐Fam in light of participant feedback and working group discussion. The CPOT‐Fam was restructured in a flow‐diagram format and dimensions were modified to include graphics and reflect simplicity. The research lead then circulated a draft of the modified CPOT‐Fam to subworking group members through email and requested any suggestions for further improvement. The research lead discussed all comments received with senior researchers and the CPOT‐Fam was revised until a version ready for pilot testing was established (shown in Supporting Information: Appendix 2).

## 4 DISCUSSION AND CONCLUSION

### 4.1 Discussion

In this study, the CPOT was adapted for family caregiver use (to create the CPOT‐Fam) and tested preclinically in preparation for clinical pilot testing. Preclinical testing of the CPOT‐Fam showed strong agreement (ICC >0.80) between reference scores and study participant scores for all dimensions of the tool. Preclinical test participants described the CPOT‐Fam as simple, easy‐to‐use, and valuable in providing structure to assessing pain in a loved one. Preclinical testing also showed that while the CPOT‐Fam was viewed favorably by most study participants (19 instances of positive sentiment of 28 total instances), opportunities for further refinement exist. The refinements were necessary to address: (1) agreement between CPOT‐Fam participant and reference scores being lower than anticipated for the *body movements* dimension, (2) qualitative feedback showing that participants prefer increased graphics and simpler language used to describe scoring options, and (3) participants disclosing indicators of pain important to them that were not currently part of the CPOT‐Fam scoring criteria.

The practice of having family caregivers assess pain in the ICU remains uncommon, with very few studies describing proxy pain assessment in critically ill patients.^2^, ^20^, ^21^ Of the sparse literature published on this topic, a multisite study with a large sample size (*n* = 2645) on family caregivers assessing pain patients including ICU patients showed that family caregivers can accurately estimate the presence of pain in patients in 73.5% of the time, and determine exact level of pain 53.0% of the time.^20^ This study also showed that family members identified the presence of pain (particularly at higher levels of pain) more accurately in comparison to the absence of pain.^20^ This study was complemented by another study which showed that family caregivers' pain ratings had higher agreement (ICC = 0.43) with patient's self‐reported pain rather than ratings by physicians (ICC = 0.40) or nurses (ICC = 0.29).^2^ Both studies reported higher participation in proxy pain assessment by female family members and spouses of critically ill patients,^2^, ^20^ suggesting that certain family caregivers may be more suited to accurate proxy pain assessment than others. While these studies were conducted with patients who were able to self‐report their pain, so comparisons with their family caregivers' pain assessment could be made, it is reasonable to imagine that family caregivers' familiarity with a patient's individualistic pain behaviors could contribute to clinicians' pain assessment for noncommunicative patients.

While family caregivers could be well‐equipped to assist in pain assessment of ICU patients, others could benefit from structure (e.g., a tool) to assist them in this endeavor. In a study of the relevance of pain behaviors in traumatic brain injury patients in ICU, family caregivers rated facial expressions such as tearing or brow lowering and body movements such^22^ as attempting to touch the pain site to be most representative of the patients' pain.^5^ However, while family caregivers were able to pinpoint specific behaviors of pain in a patient, two studies have found that family caregivers tend to slightly overestimate pain in their loved ones (16.8% of the time in one study),^20^ particularly in ventilated patients.^21^ This could be due to anxiety and distress arising from witnessing pain in a loved one.^22^ Information around the emotional effect of assessing pain on family caregivers is limited and was not collected in our current study. The provision of a structure that family caregivers can use to identify pain in their noncommunicative loved one may be able to help family caregivers assess pain in a critically ill patient more objectively. As pain behaviors described by family caregivers in this study relate to the *facial expressions* and *body movements* dimensions in original CPOT, the tool stands a good chance of adaptability for family use. Preclinical testing of the CPOT‐Fam has shown the tool's potential for pain assessment and indicated the tool could be quite useful to pain assessment of noncommunicative patients in the ICU if pilot tested and further refined.

#### 4.1.1 Limitations

This study had several limitations. While textual feedback received on the CPOT‐Fam was largely positive, it is important to note that the demographic characteristics of the study participants (mostly male, English‐speaking, university educated) may not represent the diversity of ICU family caregivers.^5^, ^10^, ^20^, ^21^, ^22^ In addition, due to the COVID‐19 pandemic, this study was conducted largely virtually. A call for recruitment in the study was circulated through social media channels that have audiences of tech‐savvy, educated, younger individuals. Hence, most participants of this study were young (ages 20−39, 77.0%) and university‐educated (60.0%). Furthermore, this study employed a modest sample size (*n* = 30) for preclinical testing, however, the textual feedback received was comprehensive and seemed to appropriately suggest refinements for the CPOT‐Fam and determine whether the tool is suitable for clinical pilot testing. Additionally, family caregiver scoring of the CPOT‐Fam was compared to a reference standard which may be different from scores assigned by bedside nurses. Finally, the study results may have been impacted by any patients having had a prior stay in the ICU (i.e., their family may have had more exposure to the ICU environment and been able to gauge pain more comfortably than others). The effect of prior ICU exposure on CPOT‐Fam scores (if any) by families will be explored as this tool is further developed and tested in clinical circumstances.

### 4.2 Conclusions

In this study, the CPOT‐Fam was developed to provide family caregivers with a structured tool to use in pain assessment of an adult critically ill patient in the ICU. Preclinical assessment suggests that family caregivers provide similar assessments of pain using the CPOT‐Fam as reference standards. This tool requires further testing to determine feasibility, acceptability, and performance in clinical circumstances. Codesigned with partners, researchers, and clinicians, the CPOT‐Fam has the potential to improve pain detection and timely pain management as well as patient health outcomes and patient‐centered care for critically ill patients in ICU.

## AUTHOR CONTRIBUTIONS

**Anmol Shahid**: conceptualization; data curation; formal analysis; investigation; methodology; project administration; validation; visualization; writing – original draft; writing – review & editing. **Bonnie G. Sept**: conceptualization; data curation; investigation; methodology; writing – review & editing. **Shelly Longmore**: conceptualization; investigation; methodology; writing – review & editing. **Victoria S. Owen**: conceptualization; investigation; methodology; writing – review & editing. **Stephana J. Moss**: investigation; methodology; writing – review & editing. **Andrea Soo**: formal analysis; investigation; methodology; writing – review & editing. **Kirsten M. Fiest**: conceptualization; investigation; methodology; resources; supervision; writing – review & editing. **Céline Gélinas**: conceptualization; investigation; methodology; supervision; visualization; writing – review & editing. **Henry T. Stelfox**: Conceptualization; funding acquisition; investigation; methodology; project administration; resources; software; supervision; writing – review & editing. All authors have read and approved the final version of the manuscript.

## CONFLICT OF INTEREST

The authors declare no conflict of interest.

## TRANSPARENCY STATEMENT

The lead author Dr. Henry T. Stelfox affirms that this manuscript is an honest, accurate, and transparent account of the study being reported; that no important aspects of the study have been omitted; and that any discrepancies from the study as planned (and, if relevant, registered) have been explained.

## Supporting information

Supplementary information.

## Data Availability

The data that support the findings of this study are available from the corresponding author upon reasonable request. Dr. Henry Thomas Stelfox had full access to all of the data in this study and takes complete responsibility for the integrity of the data and the accuracy of the data analysis.

## References

[1] Gupta M , Sahi M , Bhargava A , Talwar V . The prevalence and characteristics of pain in critically ill cancer patients: a prospective nonrandomized observational study. Indian J Palliat Care. 2015;21(3):262‐267. 26600692 10.4103/0973-1075.164894 PMC4617031

[2] Puntillo KA , Neuhaus J , Arai S , et al. Challenge of assessing symptoms in seriously ill intensive care unit patients: can proxy reporters help? Crit Care Med. 2012;40(10):2760‐2767. 22890258 10.1097/CCM.0b013e31825b94d8 PMC3712644

[3] Gélinas C . Pain assessment in the critically ill adult: recent evidence and new trends. Intensive Critical Care Nurs. 2016;34:1‐11. 10.1016/j.iccn.2016.03.001 27067745

[4] Herr K , Coyne PJ , Ely E , Gélinas C , Manworren RCB . Pain assessment in the patient unable to self‐report: clinical practice recommendations in support of the ASPMN 2019 position statement. Pain Manag Nurs. 2019;20(5):404‐417. 31610992 10.1016/j.pmn.2019.07.005

[5] Vanderbyl BL , Gélinas C . Family perspectives of traumatically brain‐injured patient pain behaviors in the intensive care unit. Pain Manag Nurs. 2017;18(4):202‐213. 28601477 10.1016/j.pmn.2017.04.005

[6] Rajasagaram U , Taylor DM , Braitberg G , Pearsell JP , Capp BA . Paediatric pain assessment: differences between triage nurse, child and parent. J Paediatr Child Health. 2009;45(4):199‐203. 19426378 10.1111/j.1440-1754.2008.01454.x

[7] Caruso TJ , Kung TH , Good J , et al. Improving satisfaction with pediatric pain management by inviting the conversation. Joint Commission J Qual Patient Saf. 2018;44(4):227‐232. 10.1016/j.jcjq.2017.10.003 29579448

[8] Azevedo‐Santos I , DeSantana J . Pain measurement techniques: spotlight on mechanically ventilated patients. J Pain Res. 2018;11:2969‐2980. 30538536 10.2147/JPR.S151169 PMC6255280

[9] Devlin JW , Skrobik Y , Gélinas C , et al. Clinical practice guidelines for the prevention and management of pain, agitation/sedation, delirium, immobility, and sleep disruption in adult patients in the ICU. Crit Care Med. 2018;46(9):e825‐e873. 30113379 10.1097/CCM.0000000000003299

[10] Mohand‐Saïd S , Lalonde MR , Boitor M , Gélinas C . Family members' experiences with observing pain behaviors using the critical‐care pain observation tool. Pain Manag Nurs. 2019;20(5):455‐461. 31109880 10.1016/j.pmn.2018.11.001

[11] Echegaray‐Benites C , Kapoustina O , Gélinas C . Validation of the use of the critical‐care pain observation tool (CPOT) with brain surgery patients in the neurosurgical intensive care unit. Intensive Critical Care Nurs. 2014;30(5):257‐265. 10.1016/j.iccn.2014.04.002 24836539

[12] Kotfis K , Zegan‐Barańska M , Szydłowski Ł , Żukowski M , Ely WE . Metody oceny natężenia bólu u dorosłych pacjentów oddziałów intensywnej terapii—polska wersja językowa narzędzia CPOT (critical care pain observation tool) I BPS (behavioral pain scale). Anestezjologia Intensywna Terapia. 2017;49(1):66‐72.28362033

[13] Gélinas C , Joffe AM , Szumita PM , et al. A psychometric analysis update of behavioral pain assessment tools for noncommunicative, critically ill adults. AACN Adv Crit Care. 2019;30(4):365‐387. 31951666 10.4037/aacnacc2019952

[14] Shoemaker SJWM , Brach C . The patient education materials assessment tool (PEMAT) and user's guide. In: Rockville MD Abt Associates I. Agency for Healthcare Research and Quality; 2013.

[15] Shahid A , Owen VS , Sept BG , et al. Study protocol: development and pilot testing of the critical care pain observation tool for families (CPOT‐Fam). Pilot Feasibility Stud. 2022;8:147 (Accepted). 35842680 10.1186/s40814-022-01102-3 PMC9287531

[16] Kottner J , Audige L , Brorson S , et al. Guidelines for reporting reliability and agreement studies (GRRAS) were proposed. Int J Nurs Stud. 2011;48(6):661‐671. 21514934 10.1016/j.ijnurstu.2011.01.016

[17] Harvey N , Holmes CA . Nominal group technique: an effective method for obtaining group consensus. Int J Nurs Pract. 2012;18(2):188‐194. 22435983 10.1111/j.1440-172X.2012.02017.x

[18] McMillan SS , King M , Tully MP . How to use the nominal group and Delphi techniques. Int J Clin Pharmacy. 2016;38(3):655‐662. 10.1007/s11096-016-0257-x PMC4909789 26846316

[19] Landis JR , Koch GG . The measurement of observer agreement for categorical data. Biometrics. 1977;33(1):159‐174. 843571

[20] Desbiens NA , Mueller‐Rizner N . How well do surrogates assess the pain of seriously ill patients? Crit Care Med. 2000;28(5):1347‐1352. 10834677 10.1097/00003246-200005000-00015

[21] Fink RM , Makic MBF , Poteet AW , Oman KS . The ventilated patient's experience. Dimens Crit Care Nurs. 2015;34(5):301‐308. 26244246 10.1097/DCC.0000000000000128

[22] Richard‐Lalonde M , Boitor M , Mohand‐Saïd S , Gélinas C . Family members' perceptions of pain behaviors and pain management of adult patients unable to self‐report in the intensive care unit: a qualitative descriptive study. Canadian J Pain. 2018;2(1):315‐323. 10.1080/24740527.2018.1544458 PMC8730585 35005388

