# Supplementary material for: Development and preclinical testing of the critical care pain observation tool for family caregiver use (CPOT‐Fam)
Source: Health Sci Rep. 2022 Dec 9;6(1):e986. doi: 10.1002/hsr2.986 (PMC9732740; doi:10.1002/hsr2.986)
Supplement: Supplementary file 1 — Supplementary information. [file HSR2-6-e986-s001.docx]

# Appendices

**Appendix 1a.** CPOT-Fam tool (version 1), used for pre-clinical testing.

**Read the questions below and check off the observation that most closely matches yours.**

| **How is the patient breathing?**  *Answer only if the patient is breathing using a breathing tube and breathing machine.* | 🞏 Breathing comfortably  🞏 Breathing comfortably with occasional coughing  🞏 Choking or struggling to breathe |
| --- | --- |
| **Is the patient making any sounds?**  *Answer only if patient is breathing on their own, without a breathing tube and breathing machine.* | 🞏 Talking in normal or quiet voice  🞏 Sighing or moaning  🞏 Calling out or sobbing |
| **What is the patient’s facial expression?**  *Please choose from the faces^[[1]](#footnote-1)^ shown. Faces on the left side of each square are patients breathing with a breathing machine and faces on the right side of each square are patients breathing without a breathing machine.* | 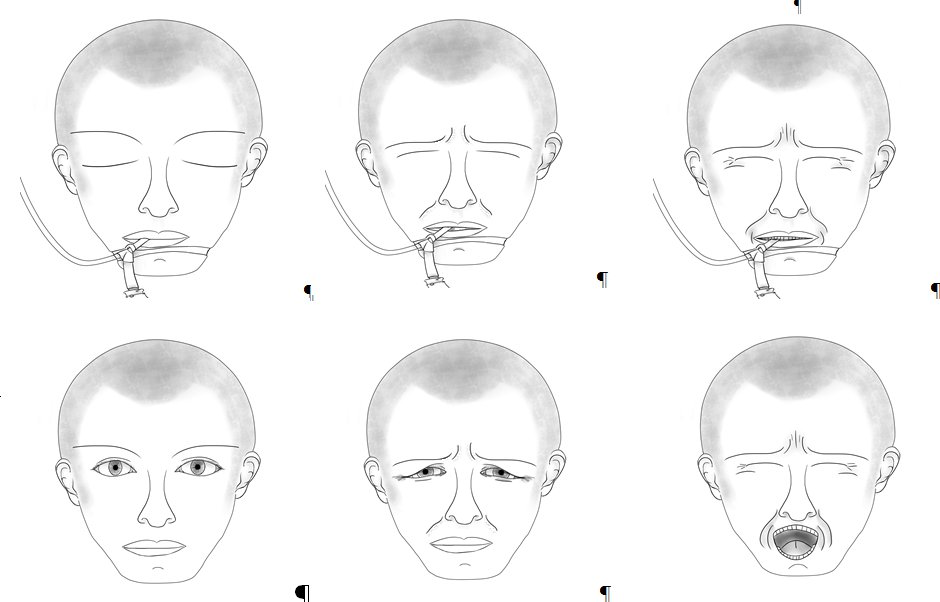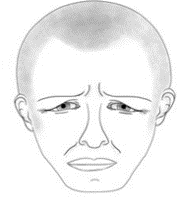    🞏 **Relaxed face**  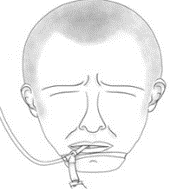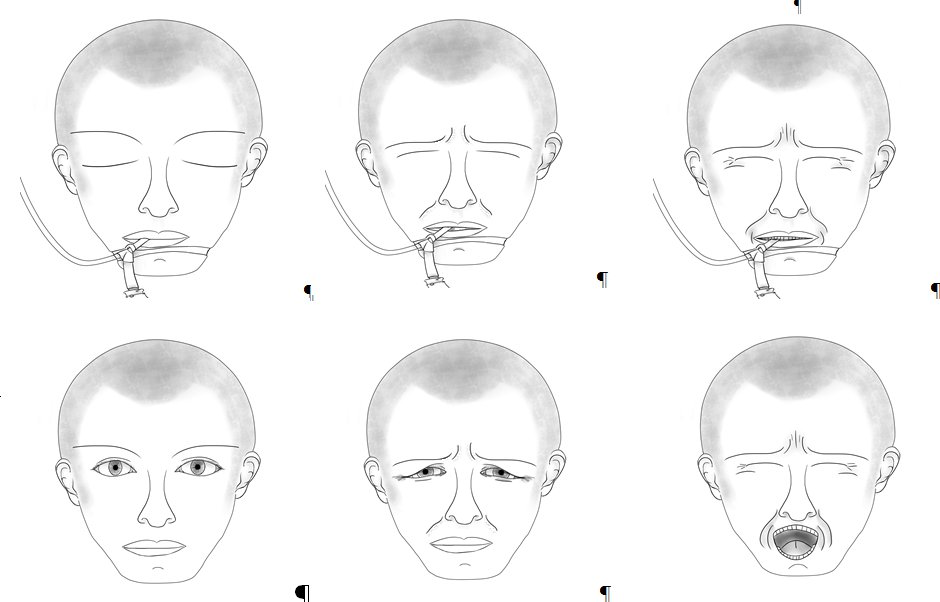  🞏 **Frowning**  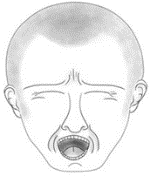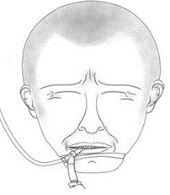  🞏 **Tense face** (clenching jaw,  uneasy, or biting on breathing tube) |
| **What are the patient’s body movements like?** | 🞏 Relaxed or comfortable (examples: lying down, sitting, or moving without pain)  🞏 Moving slowly & carefully (example: touching the site of pain)  🞏 Restless or trying to pull out tubes (examples: breathing or feeding tube, intravenous tube, catheter) |
| **Does the patient have stiff muscles?** | 🞏 Calm and relaxed (no stiffness)  🞏 Some tense or stiff muscles  🞏 Most muscles tense or stiff |

**Appendix 1b.** Links to the educational materials, presented to pre-clinical study participants.

**Pain in the intensive care unit (ICU)** – Educational video

URL - <https://vimeo.com/649227157>

**How to detect pain in the intensive care unit (ICU)** - Educational video

URL - <https://vimeo.com/649228505>

**Appendix 1c.** Sample cases (n=20) were created alongside patient partners and clinicians for participants to use with the CPOT-Fam. Select cases(A-E) used in pre-clinical testing are shown below.

**Case A  Patient:** 45-year-old man

**Reason for hospitalization:** Life threatening infection

**What you observed when you visited your loved one:**

You enter the room to visit your loved one. He is breathing through a breathing tube and his breathing seems easy. He seems to be asleep and his face appears relaxed. He is laying very still and seems calm.

**Case B  Patient:** 45-year-old man

**Reason for hospitalization:** Life threatening infection

**What you observed when you visited your loved one:**

You enter the room to visit your loved one. He is breathing through a breathing tube and seems to be struggling to breathe. He seems to be frowning. He sees you and carefully tries to adjust his body position. You touch his arm and notice it feels a bit stiff.

**Case C  Patient:** 45-year-old man

**Reason for hospitalization:** Life threatening infection

**What you observed when you visited your loved one:**

You enter the room to visit your loved one. He is breathing through a breathing tube and seems to be struggling to breathe. He seems to be clenching his jaw and his face appears tense. He seems very restless. You notice his body seems very tense.

**Case D  Patient:** 80-year-old woman

**Reason for hospitalization:** Pneumonia

**What you observed when you visited your loved one:**

You enter the room to visit your loved one. She is talking to the nurse in a normal tone. Her face seems very uneasy. She seems very restless. You notice her body seems very tense.

**Case E  Patient:** 80-year-old woman

**Reason for hospitalization:** Pneumonia

**What you observed when you visited your loved one:**

You enter the room to visit your loved one. You hear her sighing. She is frowning. She sees you and carefully tries to adjust her body position. You notice her body seems very tense.

**Appendix 2.** CPOT-Fam, after pre-clinical testing and reiterative revisions with the working group of stakeholders.


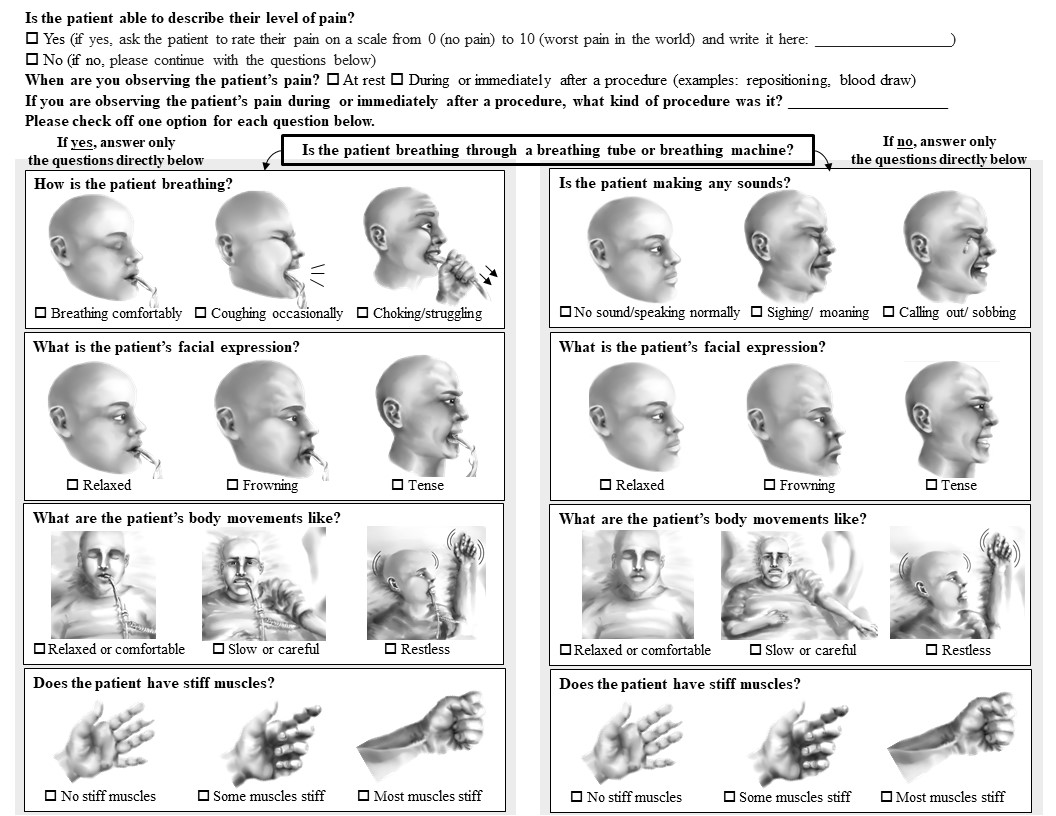


1. Illustrations of faces adapted from Gelinas et al (2013). [↑](#footnote-ref-1)
